# Multi‐Listing Among Rural Kidney Transplant Recipients: A National Cohort Study

**DOI:** 10.1111/jrh.70156

**Published:** 2026-04-30

**Authors:** Erin R. Weeda, Arwen B. L. Declan, Patricia J. Greene, Derek A. DuBay

**Affiliations:** ^1^ University of South Carolina School of Medicine Greenville Greenville South Carolina USA; ^2^ Prisma Health Upstate Greenville South Carolina USA; ^3^ Clemson University School of Health Research Clemson South Carolina USA

**Keywords:** kidney transplantation, multi‐listing, organ procurement, rural population, waiting list

## Abstract

**Purpose:**

Rural patients who qualify for kidney transplantation are less likely to undergo transplant compared to urban patients. Multi‐listing, or being added to the waiting list at more than two centers, has been shown to improve kidney transplantation rates, yet it remains a subject of debate and is understudied in rural populations. We sought to compare demographic, clinical, and donor characteristics among rural kidney transplant recipients who were on the waiting list at two or more centers (i.e., multi‐listed) versus those who were on the waiting list at one center (i.e., single‐listed) prior to transplant.

**Methods:**

This retrospective, cohort study used the Scientific Registry of Transplant Recipients (SRTR) to identify adult, first‐time, deceased‐donor kidney transplant recipients between 2019 and 2023 who resided in rural areas, as defined by Rural‐Urban Commuting Area (RUCA) Codes. Patients were divided into cohorts based on their listing status (single‐listed vs. multi‐listed) before transplant.

**Findings:**

Of the 6246 rural kidney transplant recipients included, 829 (13.3%) were multi‐listed. Compared to single‐listed recipients, multi‐listed recipients were more likely to be college‐educated (54% vs. 44%, *p* < 0.001) and employed (26% vs. 23%, *p* = 0.02); they also had a shorter duration of dialysis before transplant (33 months [interquartile range, IQR = 18–50] vs. 35 months [15–62], *p* = 0.01). However, donors for multi‐listed recipients had a higher median Kidney Donor Profile Index (50% [IQR = 25%–69%] vs. 44% [23%–66%], *p* = 0.006).

**Conclusions:**

These findings suggest that while multi‐listed rural patients possess characteristics favorable to long‐term graft success, they may receive organs from donors with less favorable profiles. Future work should focus on understanding and addressing any trade‐offs involved in multi‐listing strategies.

## 1 Introduction

Rural patients who qualify for kidney transplants are less likely to undergo transplant compared to their urban counterparts [1, 2, 3, 4]. Multi‐listing, that is, being added to the transplant waiting list at more than two transplant centers, has been shown to increase transplantation rates and may serve as a strategy to improve access for rural patients [5, 6, 7, 8, 9]. Multi‐listing allows patients the opportunity to pursue transplant at multiple transplant centers; it may expand patients’ access to available kidneys since practice patterns vary across transplant centers [10]. For instance, while studies demonstrate a predictive relationship between higher Kidney Donor Profile Index (KDPI) scores and shorter graft survival, the identification of ideal KDPI ranges continues to evolve, and centers vary in their willingness to accept kidneys from donors with high KDPI scores [10, 11, 12]. Although it appears to be beneficial for individuals, multi‐listing remains a subject of debate; the impact of multi‐listing on donor measures such as KDPI is unknown [7, 13, 14]. Further, little is known about the characteristics of rural individuals who choose to be added to the waitlist at multiple transplant centers. We sought to compare demographic, clinical, and donor characteristics among rural kidney transplant recipients who were on the waiting list at two or more centers (i.e., multi‐listed) versus those who were on the waiting list at one center (i.e., single‐listed) prior to transplant.

## 2 Materials and Methods

This retrospective cohort study compares the demographic, clinical, and donor characteristics of kidney transplant recipients in the United States who were single‐listed versus multi‐listed. This study used data from the Scientific Registry of Transplant Recipients (SRTR). The SRTR data system includes data on all donors, wait‐listed candidates, and transplant recipients in the United States, submitted by the members of the Organ Procurement and Transplantation Network (OPTN). The Health Resources and Services Administration (HRSA), US Department of Health and Human Services, provides oversight to the activities of the OPTN and SRTR contractors. The study was approved as exempt by our center's institutional review board (IRB; February 2025, Project Number: 2277687‐1).

We identified adult, deceased‐donor, solitary kidney transplant recipients who were added to the kidney transplant waiting list between January 1, 2019 and December 31, 2023. Only those who underwent their first transplant were included. The data set was limited to individuals residing in rural areas, using Rural‐Urban Commuting Area (RUCA) Codes to define rural locations [15, 16].

Based on dates from the transplant waiting list, the population of transplant recipients was separated into two cohorts: those listed at a single center (i.e., single‐listed) and those simultaneously listed at two or more centers (i.e., multi‐listed). Donor and recipient characteristics were compared between cohorts. Characteristics evaluated came from variables defined within the SRTR dataset. Thus, the variables are primarily from data that transplant centers and organ procurement organizations report to the OPTN at the time of listing, transplant, and follow‑up. Time on dialysis was calculated as the interval between dialysis initiation and the date of kidney transplantation. High‐volume centers were defined as centers in the highest quartile when the data were split into quartiles based on the number of recipients per center. Regions were defined based on OPTN regions [17]. These regions, which are used mainly for governance and communication, were adopted to maintain consistency with the transplant literature. To mitigate any risk of reidentification due to small cell sizes, smaller regions were combined with larger neighboring regions. KDPI was dichotomized at 35%, consistent with prior literature that indicates that similar thresholds serve as predictors of key post‑transplant outcomes, including kidney function and graft survival [18, 19, 20, 21].

Categorical data are presented as numbers and percentages and are compared between cohorts using chi‐square and Fisher's exact tests. Continuous data are presented as medians with interquartile ranges and are compared between cohorts using Mann–Whitney *U* tests. *p*‐values less than 0.05 were considered statistically significant. Statistical analyses and database management were conducted in SPSS Version 30 (IBM Corp., Armonk, N.Y., USA) and SAS Version 9.4 (SAS Institute Inc., Cary, N.C., USA).

## 3 Results

We identified 6246 rural kidney transplant recipients for inclusion in our study. A total of 829 (13.3%) were on the waiting list at two or more transplant centers prior to transplant. Recipient characteristics in those who were multi‐listed versus single‐listed prior to transplant are shown in Table 1. Multi‐listed recipients were more likely to be male, college‐educated, and employed. Multi‐listing differed among geographical regions, and a higher proportion of black individuals were multi‐listed. Multi‐listed individuals had a shorter median time on dialysis prior to transplant. Multi‐listed patients were not statistically more likely to get transplanted at high‐volume kidney transplant centers (71% vs. 70%, *p* = 0.37).

**TABLE 1:** Characteristics of rural kidney transplant recipients who were multi‐listed prior to transplant.

Factor	Multi‐listed *N* = 829 *n* (%)	Not multi‐listed *N* = 5417 *n* (%)	*p*
Age, years, median (IQR)	57 (46–66)	56 (45–65)	0.06
Male	516 (62)	3114 (58)	0.01
BMI, kg/m^2^, median (IQR)	29.3 (25.5–33.0)	29.5 (25.5–33.8)	0.17
Race			
White	521 (63)	3652 (67)	< 0.001
Black	272 (32)	1435 (27)	
Some college or more	446 (54)	2370 (44)	< 0.001
Employed	217 (26)	1219 (23)	0.02
Transplanted at a high‐volume center^a^	590 (71)	3773 (70)	0.37
Region of transplant center^b^			
Region 1 and 2	73 (9)	370 (7)	< 0.001
Region 3 and 4	259 (31)	1324 (24)	
Region 5, 6, and 7	67 (8)	946 (17)	
Region 8, 9, and 10	163 (19)	1418 (26)	
Region 11	266 (32)	1354 (25)	
Diabetes	343 (41)	2306 (43)	0.52
Time on dialysis prior to transplant, months, median (IQR)	33 (18–50)	35 (15–62)	0.01

Abbreviations: BMI, body mass index; IQR, interquartile range.

^a^ Defined as a center in the highest quartile when the data were split into quartiles based on the number of recipients per center.

^b^ Defined based on Organ Procurement and Transplantation Network classifications. Region 1 includes Connecticut, Maine, Massachusetts, New Hampshire, Rhode Island, and Vermont; Region 2 includes Delaware, Maryland, New Jersey, Pennsylvania, West Virginia, and Washington D.C.; Region 3 includes Alabama, Arkansas, Florida, Georgia, Louisiana, Mississippi, and Puerto Rico; Region 4 includes Oklahoma and Texas; Region 5 includes Arizona, California, Nevada, New Mexico, and Utah; Region 6 includes Alaska, Hawaii, Idaho, Montana, Oregon, and Washington; Region 7 includes Illinois, Minnesota, North Dakota, South Dakota, and Wisconsin; Region 8 includes Colorado, Iowa, Kansas, Missouri, Nebraska, and Wyoming; Region 9 includes New York and western Vermont; Region 10 includes Indiana, Michigan, and Ohio; and Region 11 includes Kentucky, North Carolina, South Carolina, Tennessee, and Virginia.

Table 2 shows donor characteristics for those who were multi‐listed versus single‐listed prior to transplant. The median KDPI was higher for multi‐listed individuals (50% [IQR = 25%–69%] vs. 44% [23%–66%], *p* = 0.006). Multi‐listed patients were less likely to receive a donor kidney with a KDPI ≤ 35 (34% vs. 40%, *p* = 0.002). Donors for multi‐listed recipients were more likely to meet the expanded donor criteria (18% vs. 15%, *p* = 0.01) and have active hepatitis C virus (17% vs. 13%, *p *< 0.001).

**TABLE 2:** Donor characteristics for rural kidney transplant recipients who were multi‐listed prior to transplant.

Factor	Multi‐listed *N* = 829 *n* (%)	Not multi‐listed *N* = 5417 *n* (%)	*p*
KDPI, %, median (IQR)	50 (25–69)	44 (23–66)	0.006
KDPI ≤ 35%	282 (34)	2144 (40)	0.002
Meets expanded donor criteria for kidney^a^	151 (18)	808 (15)	0.01
Serum creatinine, mg/dL, median (IQR)	0.9 (0.66–1.44)	0.9 (0.66–1.40)	0.38
Serum creatinine > 1.5 mg/dL	193 (23)	1171 (22)	0.28
Age, years, median (IQR)	40 (29–52)	40 (29–51)	0.65
Age ≥ 60 years	88 (11)	493 (9)	0.16
Race			
White	679 (82)	4507 (83)	0.29
Black	125 (15)	743 (14)	
BMI, kg/m^2^, median (IQR)	27.9 (23.7–33.0)	27.6 (23.6–32.9)	0.43
Hypertension	276 (33)	1633 (30)	0.07
Diabetes	94 (11)	501 (9)	0.06
Death resulting from stroke	168 (20)	1177 (22)	0.34
Donor hepatitis C positive	142 (17)	628 (12)	< 0.001

Abbreviations: BMI, body mass index; IQR, interquartile range; KDPI, Kidney Donor Profile Index.

^a^ Defined as donors ≥ 60 years of age or 50–59 years of age with two or more of the following: hypertension, death from stroke, serum creatinine > 1.5 mg/dL.

## 4 Discussion

In this national cohort study of rural kidney transplant recipients, 13.3% were listed at two or more transplant centers prior to transplantation. Consistent with studies evaluating both rural and urban populations, multi‐listed patients were more likely to have sociodemographic characteristics that typically predict better health outcomes, such as education and employment, and they experienced shorter dialysis times prior to their transplant [14]. However, a higher median KDPI, which is known to be associated with shorter graft survival [11], was observed for multi‐listed individuals, and they were more likely to receive organs from donors who had active hepatitis C virus.

Like our own analysis, several studies have evaluated factors associated with multi‐listing among transplant recipients [5, 6, 7, 8, 9]. A notable finding of the previous literature is that higher educational attainment is consistently one of the strongest predictors of multi‐listing [5, 8, 9]. The relationship has persisted across several allocation eras in the setting of kidney transplant. Earlier work spanning 1995–2000 showed that lower educational attainment was independently associated with lower odds of multi‐listing in kidney transplant candidates [5]. Similarly, an analysis of kidney transplant candidates from 2010–2017 showed that higher educational attainment was associated with multi‐listing [9]. These findings are consistent with our own analysis, where multi‐listed rural transplant recipients were more likely to have a higher educational status, as indicated by completion of at least some college. The relationship between education and multi‐listing could be explained by several factors. First, it is possible that higher educational attainment correlates with more financial resources, which may remove barriers to multi‐listing, like travel costs, obtaining time off from work, and the cost of multiple transplant evaluations. Moreover, higher educational attainment may correlate with greater health literacy. Patients with greater health literacy may be better positioned for multi‐listing because it requires comprehension and navigation of complex processes (e.g., organ allocation rules, differences across transplant centers). Similarly, multi‐listing requires coordinating multiple evaluations and facilitating the sharing of records from transplant workups. Of note, our study differs from the aforementioned prior studies in that we additionally examined donor characteristics; this allows us to also assess organ quality, which we observed to be less favorable in those that were multi‐listed. If future studies consistently demonstrate that multi‐listing is linked to receiving organs of lower quality, patients may begin factoring this risk into their decisions about listing at multiple transplant centers. Doing so would demand a high level of health literacy to navigate the associated trade‐offs and could further influence the demographic and clinical characteristics of transplant recipients.

Previous studies suggest that multi‐listing may help patients access transplants more quickly [5, 6]. As mentioned above, our findings indicate that multi‐listing may not equate to receiving higher quality donor organs among rural individuals. Taken together, this may reflect that there is prioritization of time‐to‐transplant by the center and/or the patient, even if it means accepting a lower‐quality donor organ. Previous studies have shown substantial variation in KDPI‐ and organ‐acceptance behavior of kidney transplant centers and clinicians [12, 22, 23]. By being listed at two or more transplant centers, a patient may be increasing their chances of being listed at a center where higher KDPI thresholds are used. The centers accepting higher KDPI thresholds would be expected to make an offer to transplant an organ first more often, as they are using a wider pool of organs overall. This may partially explain our finding of higher KDPIs among individuals who were multi‐listed. It is interesting to speculate on the shared organ acceptance decision‐making process that results in a more aggressive phenotype for multi‐listed patients, as evidenced by a higher donor KDPI.

Our work focused on a group of patients (i.e., rural individuals) who have historically had lower rates of kidney transplantation [1, 2, 3, 4]. Kidney transplant remains the most impactful tool for improving quality and quantity of life for patients with end‐stage renal disease [24]. Thus, interventions that increase these rates and prioritize optimal outcomes for rural kidney transplant candidates are desperately needed. Our findings suggest KDPI trade‐offs should be considered if multi‐listing is used as a strategy to reduce time‐to‐transplant among rural individuals. Interventions may be useful to better help patients and clinicians make these challenging decisions. For example, decision support tools could be implemented to help patients and clinicians navigate the complex trade‐offs between receiving a timely transplant and the anticipated post‐transplant outcomes based on donor organ quality [12]. Policymakers should also aim to strike an appropriate balance between time‐to‐transplant and donor organ quality. For instance, strategies that incentivize both shorter wait times and improved post‐transplant outcomes, including long‐term (i.e., > 1 year) graft survival, should be prioritized.

To be listed at multiple centers, transplant candidates generally would need to live within a feasible distance of multiple transplant centers. Geographic proximity to a transplant center, or the ability to overcome distance to arrive on‐site on short notice, is effectively a prerequisite to listing at a transplant center [25]. It is thus possible that single‑listed patients are not choosing to remain single‑listed but are structurally unable to pursue multi‐listing due to where they live. This may especially be true for the rural transplant candidates in our study. As a result, it is possible that donor kidneys, which are intended to be a national resource [26], may be disproportionately benefiting candidates who live in favorable locations. While we believe that geographic proximity is an important factor for access to transplant centers, we cannot evaluate this further in this study due to the inherent constraints of our dataset, which did not contain highly granular location information to protect participant privacy. Future work could explore the relationship between geographic proximity and multi‐listing.

Limitations of this study include the retrospective design and reliance on registry data alone, which lack granularity on the decisions made by the patient and transplant center. In addition, this data does not reflect important variables such as health literacy or social support. Another limitation of our study relates to generalizability and selection bias. Because our cohort included only individuals who ultimately underwent transplantation, the findings are generalizable only to transplant recipients and not to broader populations such as patients on the kidney transplant waiting list or those with end‑stage renal disease who have not yet been listed. This limitation is particularly relevant given our inclusion of rural participants, as individuals residing in rural areas are less likely to be added to the transplant waiting list than their urban counterparts [27].

In conclusion, our findings suggest that while multi‐listed rural patients possess characteristics favorable to long‐term graft success, they may receive organs from donors with less favorable profiles. To more effectively optimize patient outcomes, future work should focus on understanding and addressing any trade‐offs involved in multi‐listing strategies.

## Funding

This study was funded by a seed grant from the Prisma Health Education and Research Institute (PHERI).

## Disclosure

The data reported here have been supplied by the Hennepin Healthcare Research Institute (HHRI) as the contractor for the Scientific Registry of Transplant Recipients (SRTR). The interpretation and reporting of these data are the responsibility of the author(s) and in no way should be seen as an official policy of or interpretation by the SRTR or the US Government.

## Conflicts of Interest

The authors declare no conflicts of interest.

## Data Availability

The data that support the findings of this study are available from SRTR.
